# A Case of Aspergillus Fungal Ball in a Transplant Graft Kidney

**DOI:** 10.7759/cureus.64690

**Published:** 2024-07-16

**Authors:** Balasubramaniyan T, Karthick S Subash, Bharathi Sezhian Anbuselvam, Senthilkumar R P, Jerry Joseph

**Affiliations:** 1 Nephrology, Government Kilpauk Medical College, Chennai, IND

**Keywords:** fungal ball, kidney transplant complication, graft nephrectomy, fungal bezoars, aspergilloma

## Abstract

We report a case of an Aspergillus fungal ball in a transplant graft kidney presenting as obstructive nephropathy. This is a rare manifestation considering the usual presentations of Aspergillus infection, which are pulmonary, rhino-cerebral, and disseminated forms. Imaging showed hydronephrosis with an echogenic material in the transplant renal pelvis, which was further found to be the fungal ball. The patient underwent a graft nephrectomy due to severe sepsis, and following that, his condition improved.

## Introduction

Increasingly potent immunosuppressive agents have dramatically reduced the incidence of rejection of transplanted organs while increasing patients’ susceptibility to opportunistic infections and cancer [1-3]. Kidney transplant patients are vulnerable to fungal infections because of therapeutic immunosuppression. In solid organ transplants, filamentous fungal infections are associated with high mortality and morbidity. The Aspergillus family accounts for most infections [4,5]. Most solid organ transplant recipients with Aspergillus infections have pulmonary [6], rhino-cerebral, or disseminated infections [4]. Renal aspergillosis has rarely been reported [7]. Fungal balls of the urinary tract, also known as fungal bezoars, which are thought to be composed of mycelia, mucoid debris, and fragments from papillary necrosis, are a rare manifestation [7]. We report an isolated, unusual case of an aspergilloma presenting as a fungal ball within the renal transplant graft without disseminated infection.

## Case presentation

A 31-year-old male with a history of chronic glomerulonephritis with a dialysis vintage of four months underwent an ABO-compatible, living-related renal transplant (LRRT) with his mother as the donor. Basiliximab was the induction immunosuppressive agent used, and he was maintained on standard triple immunosuppression. Due to slow graft function with a serum creatinine of 1.7 mg/dL on day seven of transplant, a graft biopsy was done, which showed features of acute antibody-mediated rejection (ABMR), which was treated with methylprednisolone pulse, three sessions of plasmapheresis (PLEX), and intravenous immunoglobulin (IVIG) and he was discharged with a serum creatinine of 1.6 mg/dL.

**Figure 1:**
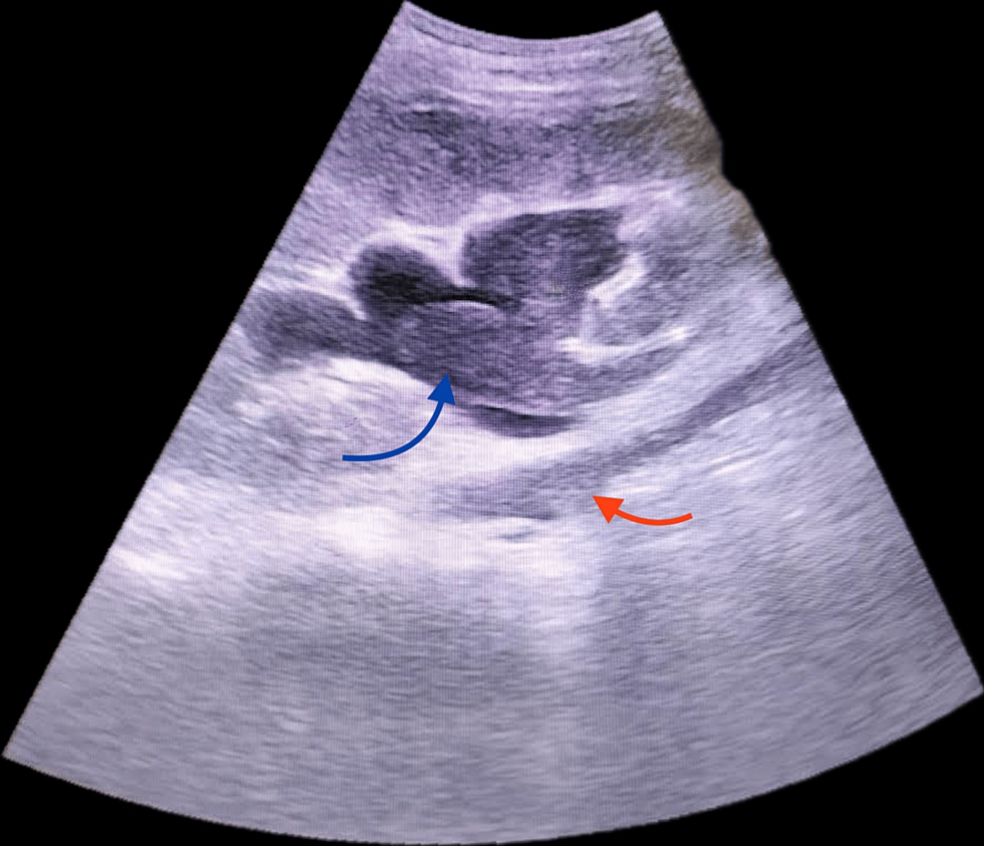
Ultrasonography of the transplant graft kidney Ultrasonography shows hydronephrosis, with an echogenic material filling the renal pelvis and continuing into the ureter (indicated by the arrow) and a collection below the ureter (indicated by the red arrow).

**Figure 2:**
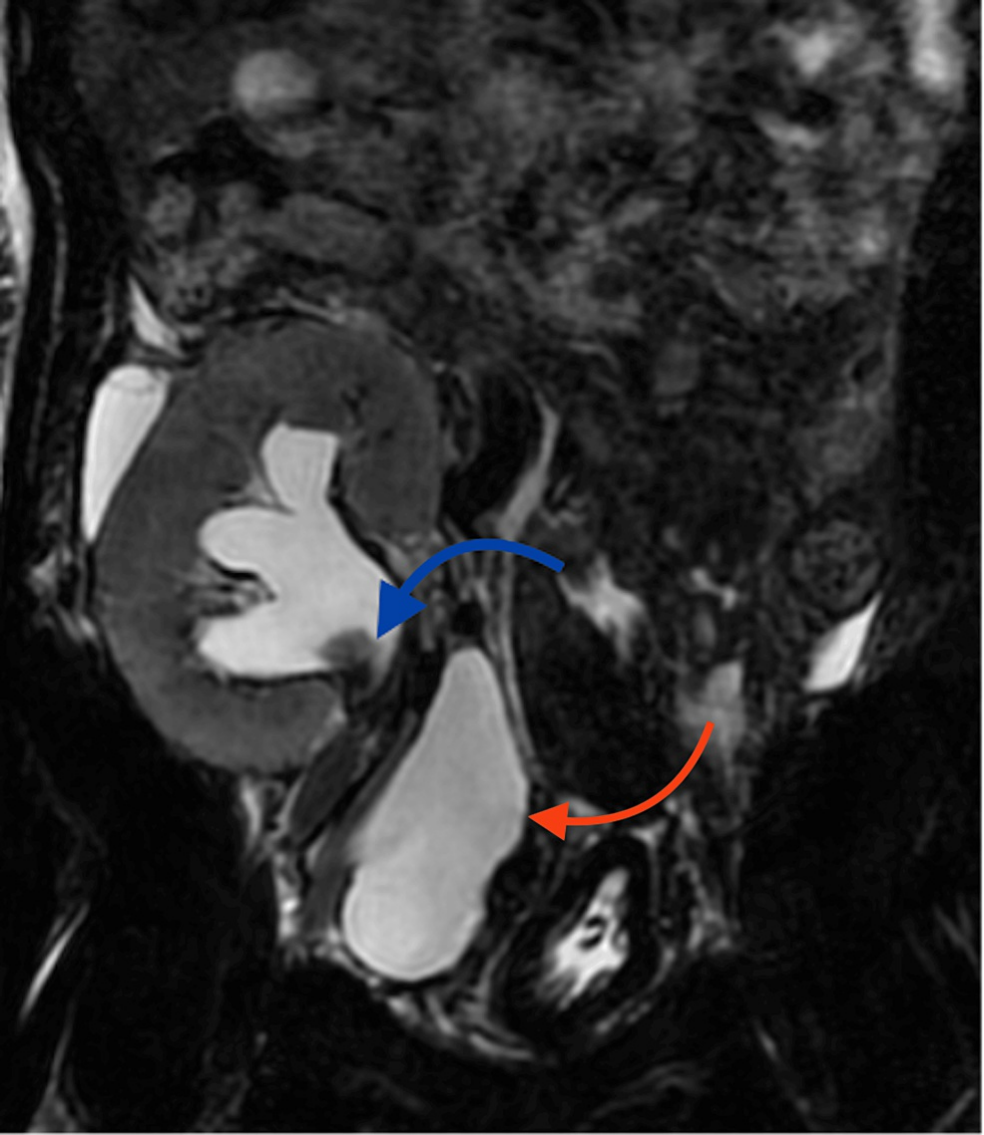
Magnetic resonance urogram The excretory phase of the magnetic resonance urogram shows hydronephrosis and a filling defect in the renal pelvis (indicated by the blue arrow). Urinoma can also be seen medially and below the transplanted kidney (indicated by the red arrow).

One and a half months post-transplant, the patient presented with anuria and acute graft dysfunction, with a serum creatinine of 6.4 mg/dL. Initial sonography suggested hydronephrosis, with echogenic material in the renal pelvis and a collection in the right iliac region (Figure 1). Magnetic resonance urography confirmed this as urinoma and also showed a filling defect in the renal pelvis (Figure 2). 

Due to acute graft dysfunction, the patient was initiated on hemodialysis. A percutaneous nephrostomy (PCN) tube and ureteral double J (DJ) stent insertion were done to relieve the pelviureteric junction (PUJ) obstruction, and a percutaneous drainage (PCD) tube was inserted to drain the perinephric collection. Post-procedure, the patient had good urine output through the PCN and urethra. A whitish fluffy material was noticed in the urine. The initial urine routine tests were normal. 

The possibilities of fungal infection and papillary necrosis were considered. However, repeated cultures of blood, urine, and the whitish material were sterile. Histopathology of the material was only suggestive of an acellular eosinophilic proteinaceous material. Four days into the hospital stay, the patient again developed reduced urine output via both routes. Repeat sonography confirmed the presence of hyperechoic material in the dilated renal pelvis, obstructing the inlets of PCN and DJ stent. PCN was reinserted. Urine output was sound for a couple of days, then again reduced. The patient was dialyzed as and when necessary.

**Figure 3:**
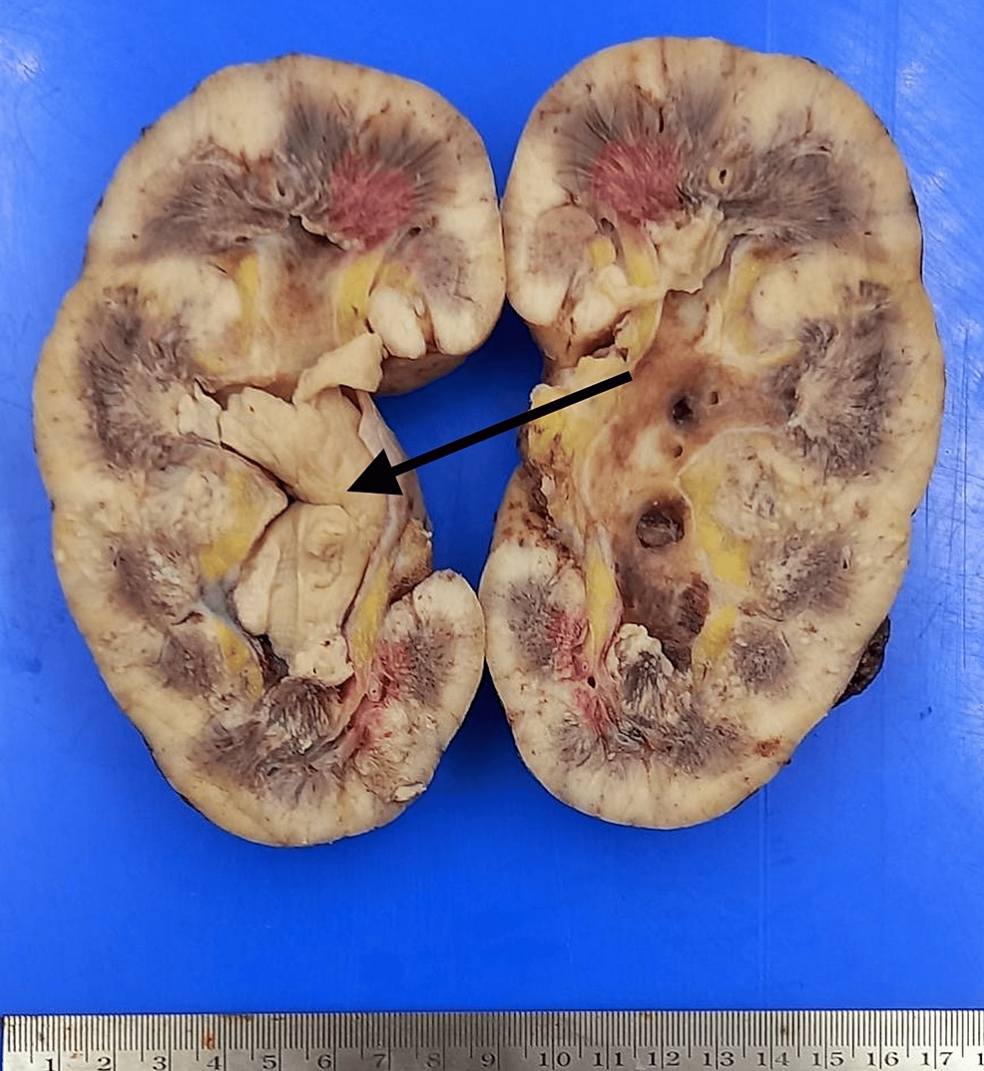
Cut-section of graft nephrectomy specimen An enlarged kidney with a cheesy whitish material fills the renal pelvis, which, on histology and culture, was diagnosed as an Aspergillus fungal ball.

The repeat urine routine suggested plenty of pus cells and a field full of RBCs. The patient also developed abdominal distention and right lower limb edema because of the increasing collection in the right iliac region. Despite being started with empirical IV antibiotics at the time of hospital admission, the patient progressed into frank sepsis. A graft nephrectomy was done on the 17th day of admission. The graft kidney was grossly enlarged, and the PCS was dilated and filled with a white cheese-like material (Figure 3).

**Figure 4:**
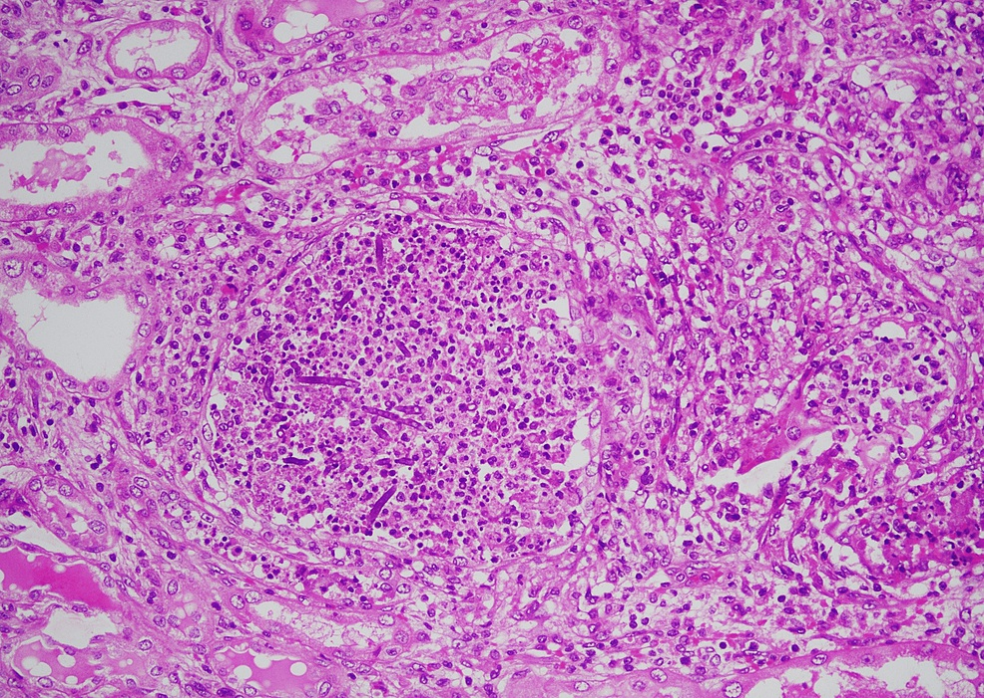
Histopathology of the renal parenchyma Light microscopy shows microabscesses composed of a focal collection of neutrophils. Numerous septate fungal hyphae are noted in those areas.

**Figure 5:**
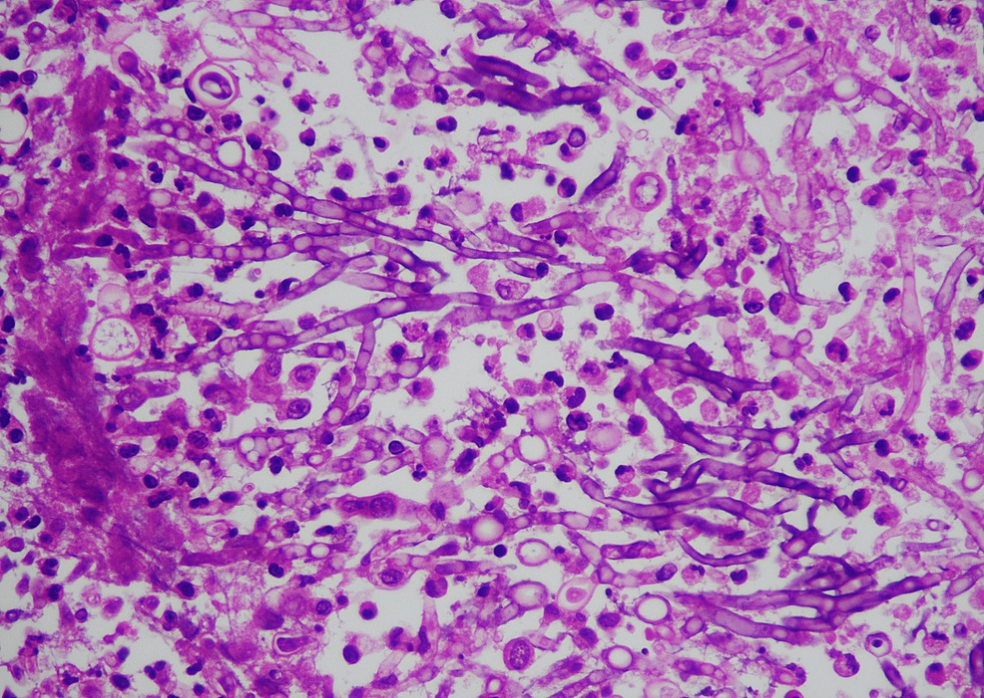
Histopathology of the cheese-like material within the renal parenchyma Light microscopy showing septate fungal hyphae with acute angle branching suggestive of Aspergillus species.

Histopathological examination confirmed the presence of Aspergillus, which had invaded the kidney and ureter (Figures 4, 5). Culture from graft bits and the whitish material from the graft pelvis grew amphotericin-resistant *Aspergillus flavus*, sensitive to voriconazole, and multidrug-resistant Acinetobacter. The patient was started with oral voriconazole and appropriate antibiotics. With the infection's focus removed and adequate antimicrobials, the patient improved and was discharged on maintenance hemodialysis.

## Discussion

It is rare for isolated aspergilloma to occur within a renal transplant graft without a systemic infection, as described in the case. There has been a dramatic increase in the incidence of aspergillosis in the last few decades, and the mortality in the transplant population with aspergillosis is around 65-92% [8]. Several mechanisms of transplantation-related transmissions are possible: transmission from an infected donor, contamination at the time of organ harvesting, preservation, or storage, or contamination at the time of transplantation [9]. Ours is a live transplantation; preservation and storage of the organ were not involved. The donor was not infected, perfusion fluid was sterile, and cultures from our transplant theatre were also sterile. 

Increased immunosuppression, multiple organ transplants, and environmental factors are some risk factors involved in this increased incidence of Aspergillosis in post-transplant patients [4]. It is noteworthy to mention that during the time that our patient underwent transplant, construction was taking place on the hospital premises. It has been shown in the past that the presence of Aspergillus species in the environment is the most critical factor in nosocomial invasive aspergillosis [4,10]. Also, around the same time, there was an outbreak of aspergillosis in our renal transplant unit, with three patients developing different forms of invasive aspergillosis. This, along with the fact that construction work was happening in our hospital, points to the fact that environmental factors might have played a significant risk factor in our case, along with the heightened immunosuppression due to recent ABMR treatment.

There should be a higher index of suspicion for fungal urinary tract infection in patients presenting with obstructive nephropathy with echogenic material in the transplant renal pelvis in the early post-transplant period, and there should be a lower threshold for starting treatment with antifungals, especially newer-generation azoles.

## Conclusions

Therapeutic immunosuppression renders kidney transplant patients vulnerable to fungal infections. In solid organ transplants, filamentous fungal infections, especially Aspergillus infections, are associated with high mortality and morbidity. In patients presenting with obstructive nephropathy with echogenic material in the transplant renal pelvis in the early post-transplant period, there should be a lower threshold for starting treatment with antifungals.
